# Research on the promotion mechanism of blended ideological and political courses on college students’ psychological capital and happiness under the background of educational digitization

**DOI:** 10.3389/fpsyg.2026.1788630

**Published:** 2026-04-10

**Authors:** Jiarui Zhao, Li Yang

**Affiliations:** School of Education Science, Harbin Normal University, Harbin, China

**Keywords:** blended learning, ideological and political courses, learning engagement, psychological capital, subjective well-being

## Abstract

**Background:**

With the advancement of educational digitalization, blended learning has become a dominant instructional approach in higher education. However, the psychological mechanisms through which blended learning promotes students’ positive psychological outcomes remain insufficiently examined. Grounded in positive psychology and psychological capital theory, this study conceptualizes blended ideological and political courses as a potential positive psychological intervention and explores how blended learning experiences influence college students’ psychological capital and subjective well-being.

**Methods:**

A cross-sectional survey was conducted among 368 undergraduate students enrolled in blended ideological and political courses at two Chinese universities. Validated instruments were used to assess perceived blended learning quality, learning engagement, psychological capital, and subjective well-being. Structural equation modeling and bootstrap mediation analyses were employed to test a chain mediation model.

**Results:**

Perceived blended learning quality significantly and positively predicted learning engagement, which in turn positively predicted psychological capital. Psychological capital was further positively associated with subjective well-being. Mediation analyses showed that learning engagement partially mediated the relationship between blended learning quality and psychological capital, while psychological capital partially mediated the relationship between learning engagement and well-being. A significant sequential mediation effect was also identified.

**Conclusion:**

This study reveals a psychological pathway through which blended learning experiences are transformed into psychological capital and enhanced well-being. The findings extend psychological capital theory in digital learning contexts and highlight learning engagement as a key psychological mechanism for promoting student well-being.

## 1 Introduction

At present, emerging intelligent technologies represented by artificial intelligence, big data, and cloud computing are accelerating a profound transformation of higher education toward digital intelligence in China (Yuan et al., 2024). Within this context, blended teaching—integrating online learning with face-to-face instruction—has become a core pedagogical paradigm rather than a simple technological supplement. Its essential value lies in the systematic reconstruction of teaching philosophy, instructional structure, and evaluation mechanisms, with the aim of cultivating learners who can adapt to the complex cognitive, emotional, and social demands of the digital era. As an important institutionalized course system in Chinese universities, ideological and political theory courses are likewise confronted with the imperative of pedagogical innovation under digital transformation (De Moortel et al., 2024; Zhou and Wang, 2026). The adoption of blended teaching modes has introduced new vitality into these courses by expanding instructional space, enhancing interactivity, and improving students’ learning experiences through the integration of online and offline learning environments. In practice, blended ideological and political courses typically combine asynchronous online learning activities—such as digital lectures, multimedia resources, and online discussion forums—with synchronous face-to-face classroom sessions that emphasize dialogue, reflection, and collaborative learning. Through this structured integration of instructional formats, blended learning environments can provide more flexible learning pathways, increase opportunities for interaction, and create richer learning experiences compared with traditional lecture-based instruction. From the perspective of educational research, students’ perceptions of these learning experiences represent an important lens for understanding how blended learning environments may influence learners’ psychological states and engagement. Such instructional arrangements not only reshape the delivery of course content but also influence students’ motivational states and psychological engagement in the learning process.

Beyond their instructional form, ideological and political courses possess intrinsic psychological value that has not yet been fully theorized or empirically examined. From the perspective of positive psychology, education is not only a process of knowledge transmission but also an important pathway for cultivating individuals’ positive psychological resources and well-being. Positive psychology emphasizes human strengths, virtues, and flourishing, focusing on the development of positive emotions, engagement, meaning, resilience, and overall well-being (Lee Duckworth et al., 2005). In this regard, the educational objectives of ideological and political courses exhibit a high degree of conceptual alignment with the core concerns of positive psychology. Through systematic instruction in Marxist theory, socialist values, and narratives of collective struggle and social responsibility, these courses guide students to construct coherent meaning frameworks and stable value identities. Such processes may foster students’ sense of purpose, hope, optimism, and psychological resilience—key components of psychological capital—by situating personal development within broader social and historical contexts. Moreover, the pedagogical design of blended ideological and political courses may further strengthen these psychological outcomes. For instance, online self-paced learning modules allow students to regulate their learning progress and repeatedly review complex theoretical content, which may enhance their sense of mastery and self-efficacy. Interactive classroom discussions and collaborative learning activities provide opportunities for students to connect theoretical knowledge with real-life experiences, thereby promoting meaning construction and psychological engagement. Through continuous feedback, reflective activities, and iterative learning tasks, students may gradually develop resilience and optimism when facing academic challenges. Importantly, these psychological outcomes are often reflected in students’ subjective learning experiences and perceptions of the learning environment, which can provide valuable insights into how blended instructional settings are associated with the development of positive psychological resources. These instructional features suggest that blended ideological and political courses may function not only as knowledge-oriented curricula but also as structured learning environments capable of cultivating positive psychological resources.

Psychological capital, typically conceptualized as a positive psychological state comprising self-efficacy, hope, resilience, and optimism, has been shown to play a crucial role in individuals’ motivation, adaptability, and well-being (Lyu et al., 2022). Prior research suggests that psychological capital is not a fixed trait but a malleable resource shaped by educational experiences and sociocultural contexts (Demerath et al., 2008; De Moortel et al., 2024). Accordingly, a well-designed blended ideological and political course may function not only as a vehicle for value education but also as a structured positive psychological intervention that contributes to the accumulation of students’ psychological capital and, ultimately, to their subjective well-being. However, despite this theoretical potential, significant gaps remain in the existing literature. First, most studies evaluating the effectiveness of ideological and political courses continue to focus on traditional outcomes such as knowledge acquisition, political identity, or moral attitudes, while psychological outcomes are often treated as secondary or incidental. Second, although a small number of studies have acknowledged the relationship between ideological and political education and psychological capital, there is a lack of empirical research that explicitly conceptualizes blended ideological and political courses as intentional intervention contexts for enhancing students’ psychological capital (Lyu et al., 2022). More importantly, the internal psychological mechanism through which blended teaching experiences influence specific components of psychological capital—and how these effects subsequently translate into students’ subjective well-being—remains insufficiently explored. The dynamic psychological process linking teaching design, learning experience, psychological resources, and well-being outcomes has yet to be systematically uncovered.

Against this backdrop, the present study adopts a perception-based survey perspective to examine how students perceive the influence of blended ideological and political courses on their psychological capital and well-being within the context of educational digitalization. Rather than focusing on objective instructional outcomes, this study emphasizes students’ subjective evaluations of blended learning experiences and seeks to clarify the internal psychological mechanisms underlying these perceptions. Specifically, this study proposes a structural relationship model in which perceived blended learning quality serves as the antecedent variable, learning engagement and psychological capital function as sequential mediators, and subjective well-being represents the outcome variable. In this model, perceived blended learning quality is expected to enhance students’ learning engagement, which in turn facilitates the development of psychological capital; psychological capital is further hypothesized to promote students’ subjective well-being. In addition, learning engagement and psychological capital are proposed to form a chain mediation pathway, through which instructional experiences are transformed into positive psychological outcomes. The theoretical contribution of this research lies in its attempt to reconceptualize blended ideological and political courses as a form of positive psychological intervention in the digital era, thereby bridging ideological and political education with positive psychology. By explicitly specifying and empirically testing the structural relationships among blended learning quality, learning engagement, psychological capital, and well-being, this study extends the application of psychological capital theory into a culturally specific educational context and provides a process-oriented explanation of how instructional design can foster students’ psychological flourishing. From a practical perspective, the findings are expected to offer empirical guidance for optimizing the design of blended ideological and political courses, encouraging educators to integrate the cultivation of positive psychological resources into value-oriented teaching practices. In doing so, the comprehensive educational function of ideological and political courses in fostering students with firm values, strong psychological capital, and enhanced well-being can be more fully realized.

## 2 Literature review and hypotheses development

### 2.1 Research on psychological capital

Psychological capital is a positive psychological state that individuals show in the process of growth and development. It goes beyond the traditional human capital and social capital, and pays attention to the internal advantageous resources of individuals. This concept is rooted in the positive psychology movement, which was initiated by Seligman and Csikszentmihalyi, and advocated that psychology should not only focus on mental diseases, but also study the virtues and advantages that make life valuable (Seligman and Csikszentmihalyi, 2014; Gable and Haidt, 2005). Early studies were devoted to defining the connotation and goal of positive psychology, which was believed to explore and cultivate the inherent potential and strength of individuals, so as to promote the prosperity of individuals and communities (Seligman, 2002; Csikszentmihalyi and Seligman, 2000). Subsequently, the field systematically reviewed its development process and looked forward to the bright future of studying positive emotions, positive traits and positive institutions through scientific methods (Alex Linley et al., 2006; Seligman, 2019). At the level of clinical practice, positive psychology has been proved to be able to effectively improve well-being, and its intervention measures such as “gratitude visit” and “advantage utilization” have become effective tools to improve individual positive experience (Lee Duckworth et al., 2005). As a concrete construct of positive psychology in the field of organizational behavior and education, the core elements of psychological capital usually include self-efficacy, optimism, hope and resilience. Research shows that psychological capital is not fixed, it is deeply affected by social background. For example, Demerath et al. (2008) pointed out that in the identity construction of American suburban high school students, the dimension of psychological capital is closely intertwined with neoliberalism ideology. The research of de moortel et al. (2024) further revealed that there is a correlation between psychological capital and social class inequality. Individuals of high social class usually have richer psychological capital, which provides a new psychological perspective for understanding social inequality. These studies have laid the foundation of psychological capital as a measurable and exploitable key positive psychological resource, and provided a theoretical basis for the follow-up study of its role in educational intervention.

### 2.2 Research on hybrid ideological and political course under the background of digital intelligence

The wave of Educational Digital Intelligence provides unprecedented technological empowerment and development opportunities for the reform and innovation of Ideological and political courses in Colleges and universities. The current research generally believes that the integration of AI, big data and other digital intelligence technologies into Ideological and political teaching is an inevitable trend to improve its timeliness and effectiveness. Many scholars have discussed the specific path and logic of digital intelligence technology in optimizing the teaching of Ideological and political course. For example, Dong and Dong (2023) studied how to use artificial intelligence technology to optimize ideological and political education in Colleges and Universities under the guidance of Ideological and political thinking in the curriculum. Liu et al. (2025) more specifically discussed the practice path of digital intelligence technology enabling ideological and political education, emphasizing the need for deep integration of technology application and educational objectives. At the macro level, Gao (2024) systematically discussed the triple logic of the integration of digital intelligence technology from the perspective of mainstream ideology construction, and clarified its necessity and strategy. In practice, blended teaching has become the core carrier of digital intelligence transformation. Zheng (2024) proposed the idea of constructing the ideological and political education system in Colleges and Universities Based on intelligent digital education, aiming to create a more intelligent and personalized learning environment. The research of Du et al. (2023) further shows how the ideological and political education integrated with AI big data can achieve mode innovation under the support of wireless network, such as accurate diagnosis of learning situation and dynamic adjustment of teaching content through data analysis. Lv (2025) extended the perspective to the optimization of the “Ideological and political parenting” model, and discussed how the digital intelligence empowerment can more comprehensively affect the growth ecosystem of students. These studies jointly point out that the hybrid ideological and political course in the context of digital intelligence is no longer a simple combination of online and offline teaching forms, but a new education form driven by data and supported by intelligent technology, which can achieve precise teaching, in-depth interaction and whole process evaluation. Its ultimate goal is to enhance the attractiveness, appeal and teaching effectiveness of the ideological and political course.

### 2.3 Research on ideological and political

Although the empirical research on the relationship between Ideological and political courses and college students’ well-being is still in its infancy, the existing literature has revealed the potential relationship between the two from multiple perspectives, in which psychological capital is regarded as a key intermediary bridge. Some studies directly focus on the impact of Ideological and political course on students’ psychological level. Yu (2021) made a dynamic analysis of the psychological state of College Students’ Ideological and political course, suggesting that the course participation may cause positive changes in students’ psychological state. More studies focus on the relationship between Ideological and political courses and the core positive psychological construct of psychological capital. The research of Fu (2020) and Fu (2019) both discussed the influence of Ideological and political education on the development of College Students’ psychological capital and the correlation between the two, which provided preliminary evidence for the ideological and political course as a positive psychological intervention measure. The research title of Luo and Xiao (2020) directly points out that the development of psychological capital has a “promoting effect” on the ideological and political education of college students, which shows that there is a close symbiotic relationship between the two in reverse. These studies suggest that ideological and political course can effectively cultivate students’ self-efficacy, optimism, life hope and resilience through its content (such as ideal and belief education, struggle spirit shaping) and process (such as value guidance and meaning construction), which are the core components of psychological capital. Further research will extend this chain to more specific behavioral outcomes. Yuan et al. (2024) found that the combination of psychological capital based on deep learning and ideological and political education has a significant impact on College Students’ entrepreneurial intention. Lyu et al. (2022) also verified the positive effect of psychological capital on Teachers’ and students’ entrepreneurial performance and sports ethics under social and political education. Although the dependent variables of these studies are entrepreneurial intention or moral behavior rather than direct well-being, according to Seligman (2003)’s perma happiness theory, sense of achievement (such as entrepreneurship) and sense of meaning (such as moral improvement) are the core elements of well-being. Therefore, although the existing literature has not been directly verified, it strongly suggests a path of “Ideological and political course—psychological capital—positive behavior results (including well-being)”, which lays a solid literature foundation for this study to explore the promotion mechanism of Ideological and political course on well-being.

### 2.4 Research hypotheses

In summary, existing literature provides a multi-level theoretical basis for this study. Positive psychology and psychological capital theory have established the core variables for research, while research on digitization and blended learning has constructed modern teaching contexts. The study of the correlation between ideological and political courses and psychological capital provides preliminary evidence for the relationship between variables. However, there are significant gaps in existing research: firstly, most studies are limited to theoretical exploration and lack rigorous empirical testing of the mechanism of the effectiveness of blended ideological and political education teaching; Secondly, the logical chain between research variables is not yet complete, and a complete model from teaching input to psychological processes and then to happiness output has not been formed. Specifically, how blended learning can influence students’ immediate learning status, cultivate their long-term psychological resources, and ultimately enhance their sense of well-being has not been empirically tested. The proposed sequential relationship among the variables is theoretically grounded in the distinction between situational learning processes and relatively enduring psychological resources. Learning engagement is generally regarded as a proximal and context-sensitive learning state, reflecting students’ vigor, dedication, and absorption in ongoing academic activities (Schaufeli et al., 2002). In contrast, psychological capital represents relatively stable positive psychological resources, including self-efficacy, hope, resilience, and optimism, which are gradually developed through repeated experiences of successful learning, active participation, and adaptive coping. From this perspective, high-quality blended learning environments are expected to first stimulate students’ engagement in learning activities. Sustained engagement in learning tasks may subsequently contribute to the development of psychological capital by fostering mastery experiences, goal attainment, and positive expectations for future performance. Therefore, positioning learning engagement as preceding psychological capital is theoretically consistent with the process through which immediate learning experiences are transformed into more enduring psychological resources.

Based on the literature review and research gaps mentioned above, this study proposes a chain mediation model and aims to test the following core hypotheses:

*H1:* The quality of blended learning has a significant positive impact on college students' learning engagement;

*H2:* Learning engagement has a significant positive impact on the psychological capital of college students;

*H3:* Psychological capital has a significant positive impact on the happiness of college students;

*H4:* Learning engagement mediates the relationship between blended learning quality and psychological capital;

*H5:* Psychological capital plays a mediating role between learning engagement and happiness.

## 3 Methods

This study adopts a perception-based survey research design to examine how students perceive the influence of blended ideological and political courses on their psychological capital and subjective well-being. As the constructs investigated in this study involve subjective psychological states and learning experiences, students’ self-reported perceptions are considered an appropriate data source for capturing these phenomena. Through a questionnaire survey, the study explores the relationships among perceived blended learning quality, learning engagement, psychological capital, and subjective well-being.

### 3.1 Instructional design of the blended ideological and political course

The ideological and political course examined in this study adopted a blended learning model that systematically integrated online and face-to-face instructional activities throughout a 16-week semester. The course was “Introduction to Mao Zedong Thought and the Theory of Socialism with Chinese Characteristics,” which is a compulsory course for undergraduate students in Chinese universities. The blended instructional design aimed to combine the advantages of digital learning environments with the depth of in-person interaction, thereby enhancing students’ learning engagement and supporting the development of positive psychological resources. In terms of course structure, the blended course consisted of both asynchronous online learning and synchronous face-to-face classroom sessions. Approximately half of the learning activities were completed through online modules hosted on the Chaoxing learning platform, while the remaining instructional time involved classroom-based discussions and interactive teaching. Each weekly learning cycle generally followed a structured sequence. Students first completed online preparatory learning tasks before attending the classroom session. These tasks included watching short video lectures, reading digital learning materials, and completing brief formative quizzes designed to ensure understanding of key theoretical concepts. Online discussion forums were also used to encourage students to reflect on course topics and exchange perspectives with peers. The synchronous face-to-face sessions were designed to deepen students’ understanding through interactive learning activities. During classroom meetings, instructors organized group discussions, case analyses, and collaborative presentations related to contemporary social issues and ideological themes. These activities encouraged students to connect theoretical knowledge with real-life experiences and social contexts. Teachers also facilitated guided reflection and dialogue, allowing students to express viewpoints, question assumptions, and collectively construct meaning around course content.

Importantly, the instructional design of the blended course incorporated several pedagogical strategies that could potentially foster students’ psychological capital. First, the online self-paced learning environment allowed students to control their learning pace and repeatedly review complex theoretical materials, which may enhance their sense of competence and academic self-efficacy. Second, goal-oriented learning tasks and staged assessments were used to help students experience incremental progress during the learning process, thereby strengthening hope and motivation. Third, collaborative discussions and problem-solving activities provided opportunities for students to confront different viewpoints and engage in critical reflection, which may cultivate psychological resilience when encountering cognitive challenges. Finally, reflective assignments and discussions about social development and personal responsibility were integrated into the course to encourage students to adopt a constructive perspective toward personal growth and social participation, potentially contributing to the development of optimism. Through the systematic integration of online and offline learning activities, the blended ideological and political course created a learning environment that emphasized interaction, reflection, and active participation. These instructional features not only enhanced the quality of students’ learning experiences but also provided a context in which learning engagement could be transformed into positive psychological resources. In this way, the blended course may be perceived by students as a learning environment that supports the accumulation of psychological capital and the promotion of students’ subjective well-being.

### 3.2 Variable settings

This study used a questionnaire survey method, and all core variables were measured using mature scales from both domestic and international sources to ensure the reliability and validity of the measurement tools. As the constructs examined in this study involve students’ psychological states and learning experiences, a perception-based self-report questionnaire was considered an appropriate approach for capturing students’ subjective evaluations of the blended learning environment and their psychological outcomes. The questionnaire adopts the Likert five point scoring method (1 = “completely disagree” to 5 = “completely agree”) to measure all items uniformly. The measurement of perceived quality in blended learning draws on the theoretical framework of the DeLine&McLean information system success model and is appropriately adapted to the characteristics of blended learning. It measures from four dimensions: system quality, information quality, service quality, and fusion quality, with a total of 12 items. This measurement method has been proven effective in evaluating students’ overall perception of blended learning environments in relevant research (Dong and Dong, 2023; Liu et al., 2025). Example items include: “The online learning platform is stable and easy to use,” “The learning resources provided in the blended course are clear and helpful,” and “The integration of online and offline learning activities is well organized.” The measurement of learning engagement adopts the Chinese version of the Learning Engagement Scale (UWES-S) developed by Schaufeli et al. (2002), which includes three dimensions of vitality, dedication, and focus, with a total of nine items. It has been widely used in the field of higher education research and can effectively capture students’ psychological states during the learning process. Example items include: “I feel energized when studying this course,” “I am enthusiastic about participating in course activities,” and “I am fully concentrated when learning course content.” The measurement of psychological capital was carried out using the simplified version of the Psychological Capital Questionnaire (PCQ-12) developed by Luthans et al. (2007), which includes four core dimensions of self-efficacy, hope, resilience, and optimism, with a total of 12 items. It has shown good psychological measurement characteristics among Chinese university students (Luo and Xiao, 2020; Fu, 2020). Example items include: “I feel confident in my ability to complete challenging learning tasks,” “I can think of many ways to achieve my academic goals,” and “I usually take a positive view toward my future development.” The measurement of subjective well-being is based on the overall subjective well-being scale developed by Diener et al. (1985), which includes three subscales: life satisfaction, positive emotions, and negative emotions, with a total of 11 items. It is a classic tool for evaluating an individual’s overall well-being, with negative emotion items being processed in reverse during scoring. Example items include: “In most ways my life is close to ideal,” “I feel satisfied with my life,” and “I often experience positive emotions in my daily life.” All scales have undergone rigorous translation and backtracking procedures and demonstrated good reliability coefficients (Cronbach’s alpha>0.8) in the pre-test, ensuring their applicability to the specific context of this study.

### 3.3 Data collection

This study adopts a cross-sectional perception-based survey design and focuses on two comprehensive universities in China, one is a “Double First Class” construction university and the other is a provincial key university. The survey aimed to collect students’ perceptions of their experiences in blended ideological and political courses and their perceived psychological outcomes. The target group is undergraduate students who have officially taken the blended ideological and political course “Introduction to Mao Zedong Thought and the Theory of Socialism with Chinese Characteristics.” This sampling strategy aims to obtain samples from different academic backgrounds and school environments to enhance the generalizability of research results.

Data collection was conducted through the professional online survey platform “Wenjuanxing.” With the assistance of research assistants, the questionnaire link and QR code were distributed to students during the last class session at the end of the semester or through the course online learning platform (such as Study Pass). Before filling out the questionnaire, all participants were informed that this study was an anonymous academic research and were required to read and sign an electronic informed consent form. To encourage participation, small random incentives (digital red envelopes) were provided to students who completed the questionnaire. The data collection period lasted approximately two weeks. In total, 412 students submitted the questionnaire. After applying data quality screening procedures, 44 questionnaires were identified as invalid and removed, resulting in a final valid sample of 368 responses (effective response rate = 89.3%). The effective sample size (*N* = 368) exceeds the commonly recommended minimum sample size for structural equation modeling, providing a sufficient basis for subsequent statistical analysis.

### 3.4 Quality control and ethical considerations

To ensure data quality and ethical standards of research, the following measures were implemented in this study:

In terms of quality control, first of all, before the questionnaire was officially distributed, we conducted a small-scale pre-test (*n* = 30), fine tuned the clarity of the question expression based on feedback, and calculated that the Cronbach’s alpha coefficient of the overall scale was greater than 0.8, indicating that the scale has extremely high internal consistency reliability. Secondly, in the formal questionnaire, we have set up attention detection items (such as “Please select ‘fully agree’ to verify that you are answering the question seriously”), and questionnaires that fail to pass this item will be considered invalid. Finally, during the data cleaning stage, several criteria were applied to identify invalid responses. Questionnaires with excessively short completion times (indicating that respondents were unlikely to have read the questions carefully) were removed. In addition, responses showing clear regularity patterns—such as selecting the same option for nearly all items or exhibiting highly consistent response sequences—were excluded. These procedures were implemented to ensure the authenticity and reliability of the dataset.

In terms of research ethics, this study strictly followed relevant ethical guidelines. The research proposal was reviewed and approved by the Academic Ethics Committee of our university. All participants were fully informed of the purpose, content, anonymity and confidentiality of the data, as well as their unconditional right to withdraw at any time before completing the questionnaire. The entire data collection and analysis process was anonymous, and no personally identifiable information was collected. All data were used only for aggregated statistical analysis and were stored securely. After the completion of the study, a summary of the research findings was provided to participants to respect their right to information and acknowledge their participation.

## 4 Results

### 4.1 Reliability

Reliability and validity analysis is a statistical validation method that evaluates the scientific validity of measurement tools by examining their reliability and effectiveness. In this study, the main factors were measured in the form of scales, so testing the data quality of the measurement results is an important prerequisite for ensuring meaningful subsequent analysis. Firstly, analyze the internal consistency of each dimension using Cronbach’s Alpha coefficient reliability test method. The Cronbach’s alpha coefficient ranges from 0 to 1. The higher the coefficient value of the test result, the higher the reliability. It is generally believed that for a variable to have good reliability, the Cronbach’s Alpha coefficient must be greater than 0.7. From the Table 1, it can be seen that the Cronbach’s Alpha coefficients of each variable are all greater than the standard of 0.7, indicating that the variables have good internal consistency reliability.

**Table 1:** Reliability analysis of measurement scales.

Variables	Cronbach’s Alpha
System quality	0.882
Information quality	0.871
Service quality	0.835
Fusion quality	0.917
Vitality	0.914
Dedication	0.848
Focus	0.891
Self-efficacy	0.889
Hope	0.942
Resilience	0.938
Optimistic	0.918
Life satisfaction	0.928
Positive emotion	0.968
Negative emotions	0.913

### 4.2 Validity and common method bias test

Validity analysis is mainly conducted through methods such as KMO, Bartlett’s Sphericity test, and total variance interpretation tool for correlation testing. Exploratory factor analysis was conducted using SPSS23.0 to perform KMO and Bartlett’s Sphericity test on the scale, and the results are shown in the following table.

From the Table 2, KMO = 0.920, greater than 0.7, and Bartlett’s sphericity test value is significant (Sig. < 0.001), indicating that the questionnaire data meets the prerequisite requirements for factor analysis. Therefore, further analysis was conducted using principal component analysis for factor extraction, and common factors were extracted based on eigenvalues greater than 1. Factor rotation was performed using maximum variance orthogonal rotation for factor analysis. The analysis results are shown in the Table 3, a total of three factors were identified, with a total explanatory power of 63.750%, which is greater than 50%. This indicates that the four selected factors have good representativeness.

**Table 2:** KMO and Bartlett inspection.

Test		Value
KMO sampling suitability quantity		0.920
Bartlett Sphericity test	Approximate chi-square	1984.086
	Degree of freedom	120
	Salience	0.000

**Table 3:** Total variance explained.

Ingredient	Initial eigenvalue			Extract the sum of squared loads			Sum of squared rotational loads		
	Total	Variance percentage	Accumulated%	Total	Variance percentage	Accumulated%	Total	Variance percentage	Accumulated%
1	7.146	44.662	44.662	7.146	44.662	44.662	3.832	23.952	23.952
2	1.729	10.809	55.471	1.729	10.809	55.471	3.641	22.757	46.709
3	1.621	9.131	59.361	1.413	9.621	59.472	3.213	7.002	53.709
4	1.325	8.279	63.75	1.325	8.279	63.75	2.727	10.041	63.75
5	0.709	4.432	68.183						
6	0.624	3.899	72.082						
7	0.557	3.481	75.563						
8	0.537	3.359	78.922						
9	0.496	3.1	82.022						
10	0.474	2.961	84.983						
11	0.457	2.855	87.839						
12	0.416	2.602	90.441						
13	0.383	2.395	92.836						
14	0.346	2.159	94.995						
15	0.309	1.933	96.928						
16	0.265	1.656	98.584						
17	0.227	1.416	100						

Given that all variables in this study were measured using self-report questionnaires, common-method bias may be a potential concern. To assess this issue, Harman’s single-factor test was conducted. The results showed that multiple factors with eigenvalues greater than 1 were extracted, and the first unrotated factor explained 44.662% of the total variance, which was below the commonly accepted threshold of 50% (Table 3). Therefore, common-method bias was not likely to seriously threaten the validity of the findings in this study.

### 4.3 Discriminant validity test

To further examine the discriminant validity of the study constructs, confirmatory factor analysis (CFA) was conducted using AMOS 26.0. The hypothesized four-factor model, including perceived blended learning quality, learning engagement, psychological capital, and subjective well-being, was compared with several alternative models in which conceptually related constructs were combined. As shown in Table 4, the hypothesized four-factor model demonstrated a good fit to the data (χ^2^/df = 2.35, CFI = 0.93, TLI = 0.92, RMSEA = 0.061), and the model fit indices were significantly better than those of the alternative models (Table 4). Specifically, when learning engagement and psychological capital were combined into a single factor (three-factor model), the model fit deteriorated noticeably. The two-factor and one-factor models showed even poorer fit indices. These results indicate that the four constructs in this study are empirically distinguishable, supporting the discriminant validity of perceived blended learning quality, learning engagement, psychological capital, and subjective well-being.

**Table 4:** Confirmatory factor analysis model comparison.

Model	χ^2^/df	CFI	TLI	RMSEA
Four-factor model (BLE, UWES, PCQ, SWLS)	2.35	0.93	0.92	0.061
Three-factor model (BLE, UWES+PCQ, SWLS)	3.47	0.88	0.86	0.082
Two-factor model (BLE + UWES, PCQ + SWLS)	4.91	0.8	0.78	0.101
One-factor model (all variables combined)	6.34	0.71	0.69	0.124

### 4.4 Correlation analysis

Use Pearson correlation coefficient method to analyze the correlation between variables in each dimension. The correlation analysis results indicate that there is a statistically significant correlation between the main variables, which provides preliminary support for the research hypothesis. Firstly, there is a significant moderate positive correlation (r = 0.514, *p* < 0.001) between perceived quality of blended learning (BLE) and learning engagement (UWES), indicating that the higher students’ evaluation of blended learning, the higher their vitality, dedication, and focus in learning (Table 5). Secondly, the perceived quality of blended learning (BLE) and psychological capital (PCQ) also showed a significant positive correlation (r = 0.488, *p* < 0.001), suggesting that a high-quality teaching experience may help improve students’ self-efficacy, hope, resilience, and optimism levels. In addition, there is a strong positive correlation (r = 0.539) between learning engagement (UWES) and psychological capital (PCQ), indicating a close association between positive learning states and positive psychological resources. Finally, subjective well-being (SWLS) showed a significant positive correlation with the other three variables (BLE, UWES, PCQ), although the correlation coefficients were relatively weak, indicating an undeniable link between the development of teaching experience, learning engagement, and psychological capital and the improvement of students’ well-being. These findings preliminarily validate the correlation between constructs in the study, laying a solid foundation for further testing of the mediating role of learning engagement and psychological capital. Specifically, the significant positive relationship between perceived blended learning quality and learning engagement provides empirical support for H1, indicating that higher perceived quality of blended learning is associated with stronger student learning engagement. The significant association between learning engagement and psychological capital supports H2, suggesting that students who demonstrate higher levels of engagement in learning are more likely to develop stronger psychological capital. In addition, the positive relationship between psychological capital and subjective well-being supports H3, indicating that higher psychological capital is related to higher levels of students’ subjective well-being.

**Table 5:** Results of correlation analysis.

Variables	BLE	UWES	PCQ	SWLS
BLE	0.824			
UWES	0.514***	0.831		
PCQ	0.488***	0.539	0.86	
SWLS	0.231***	0.262***	0.134***	0.742

*** *P* ≤ 0.001.

### 4.5 Mediation analysis

To verify the existence of the mediating effect, this study employed the Bootstrap method. In Amos 26.0 software, 5,000 repeated samplings were performed on the sample data. If the calculated confidence interval does not contain zero values, it indicates that the mediating effect is significant. The final result is at a 95% confidence level, and the specific data is shown in Table 5.

#### 4.5.1 Learning engagement mediates the relationship between blended learning quality and psychological capital

The results of the table show that the overall impact of learning engagement on occupational burnout is 0.674, and its 95% confidence intervals (Bias Corrected and Perceptile methods) do not cross zero, indicating that this effect is significant. Further decomposition reveals that the indirect effect of blended learning quality on psychological capital through the “learning engagement” pathway is −0.388, accounting for 57.6% of the total effect. Within the lower and upper value ranges of Bias Corrected and Percntile95% CI, there is no zero, indicating a significant mediating effect; The direct effect value of blended learning quality psychological capital is −0.286, accounting for 42.4%, and its confidence interval has not reached zero, indicating that the direct path is also significant (Table 6). Based on this, it can be concluded that learning engagement plays a partial mediating role between blended learning quality and psychological capital, with H4 receiving support.

**Table 6:** Mediating test of learning engagement between blended learning quality and psychological capital.

Path	Estimate	SE	Bias-corrected		*p*	Percentile		*p*	(%)
			95%CI			95%CI			
			Lower	Upper		Lower	Upper		
The mediating effect of blended learning quality learning engagement psychological capital	−0.388	0.123	−0.704	−0.224	0.000	−0.697	−0.222	0.000	57.6%
Quality of blended learning—direct effect of psychological capital	−0.286	0.124	−0.485	−0.033	0.034	−0.477	−0.009	0.044	42.4%

#### 4.5.2 Psychological capital plays a mediating role between learning engagement and happiness: mediating test

The table results show that the total effect of learning engagement on happiness is 0.427, and the 95% confidence intervals (Bias Corrected and Percentile methods) do not include zero, indicating a significant overall impact. Among them, the indirect effect of work factors through “psychological capital” is 0.314, accounting for 52.3% of the total effect, and the 95% confidence interval does not cross zero; The mediating effect value of learning investment psychological capital happiness is 0.314, accounting for 52.3%. It does not include 0 in the lower and upper value ranges of Bias Corrected and Percentile 95% CI, indicating a significant mediating effect; The direct effect value of learning engagement on happiness is 0.223, accounting for 47.7%, and its confidence interval is also non-zero, indicating a significant direct effect (Table 7). Based on this, it can be concluded that psychological capital plays a role between learning engagement and happiness, and hypothesis H5 has been validated.

**Table 7:** Mediation test of work level personal level occupational burnout.

Path	Estimate	SE	Bias-corrected		*p*	Percentile		*p*	Proportion (%)
			95%CI			95%CI			
			Lower	Upper		Lower	Upper		
The mediating effect of learning investment psychological capital happiness	0.314	0.077	0.533	0.614	0.000	0.107	0.251	0.000	52.3%
Learning engagement—direct effect on happiness	0.223	0.089	0.362	0.433	0.014	0.313	0.434	0.013	47.7%

### 4.6 Summary of empirical test results

The path coefficients of the structural equation model and the results of the mediation effect test jointly indicate that all five hypotheses proposed in this study are empirically supported, and the specific verification results are summarized in Table 8.

**Table 8:** Summary of hypothesis testing results.

Number	Assuming content	Result
H1	The quality of blended learning has a significant positive impact on college students’ learning engagement;	Establish
H2	Learning engagement has a significant positive impact on the psychological capital of college students;	Establish
H3	Psychological capital has a significant positive impact on the happiness of college students;	Establish
H4	Learning engagement mediates the relationship between blended learning quality and psychological capital;	Establish
H5	Psychological capital plays a mediating role between learning engagement and happiness.	Establish

## 5 Discussion

**Figure 1:**
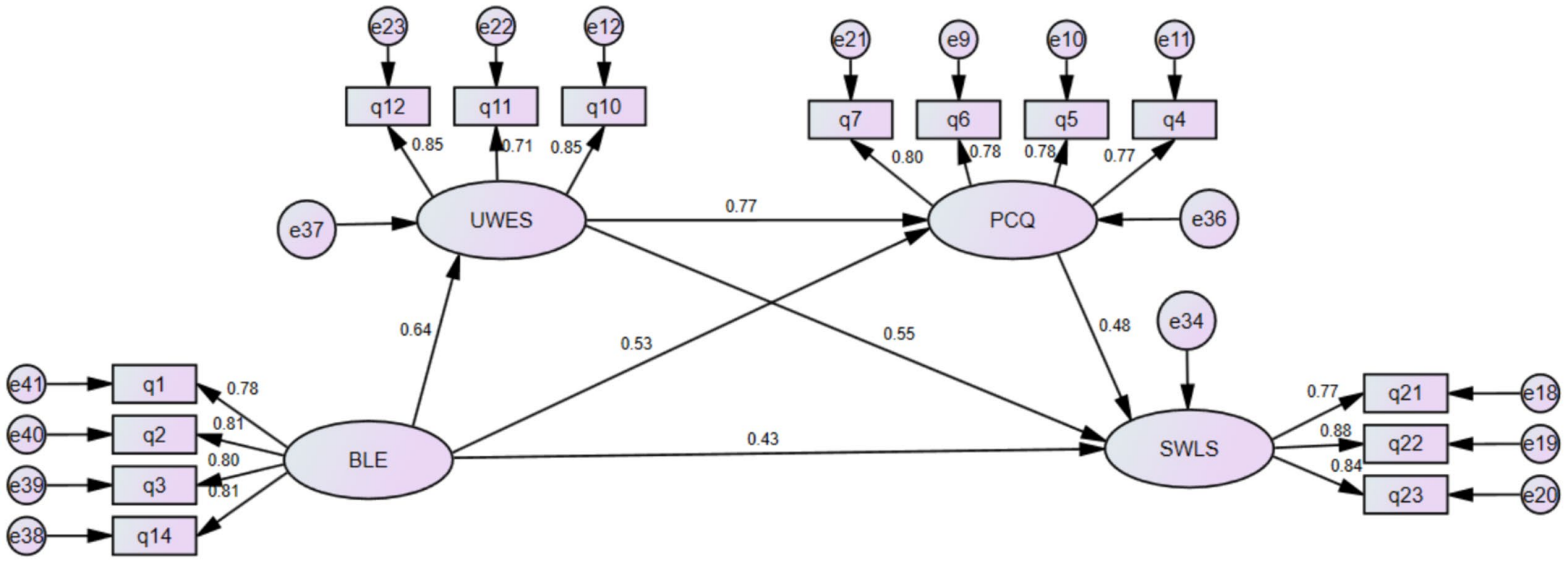
Structural relationship model.

Based on a chain mediation model, this study empirically examined the psychological mechanism through which blended ideological and political courses influence college students’ subjective well-being. The results demonstrate that blended learning quality positively predicts learning engagement, learning engagement significantly promotes psychological capital, and psychological capital, in turn, enhances students’ subjective well-being (Figure 1). Moreover, learning engagement and psychological capital jointly form a sequential mediation pathway linking blended learning quality and well-being. These findings not only validate all proposed hypotheses (H1–H5), but also provide a systematic psychological explanation for how pedagogical design in the digital era can be transformed into sustainable psychological resources and positive well-being outcomes. However, it should be noted that the findings of this study are based on cross-sectional survey data reflecting students’ perceptions of their learning experiences. Therefore, the results indicate significant associations among the variables rather than definitive causal relationships. The interpretations and practical implications discussed below should be understood within this methodological context. The discussion below elaborates on these findings from three interrelated perspectives.

### 5.1 Blended learning as a multi-pathway mechanism for the accumulation of psychological capital

One of the most important findings of this study is that blended learning quality exerts both direct and indirect effects on students’ psychological capital, with learning engagement serving as a key mediating mechanism. Specifically, high-quality blended learning significantly enhances students’ vitality, dedication, and absorption in learning, which subsequently promotes the development of self-efficacy, hope, resilience, and optimism. This result is consistent with previous research emphasizing the malleability of psychological capital and its sensitivity to educational contexts (Demerath et al., 2008; De Moortel et al., 2024; Wang, 2025), and further extends these findings to the context of blended ideological and political education.

From a theoretical perspective, this mechanism can be effectively explained by Fredrickson’s broaden-and-build theory of positive emotions. According to this theory, positive learning experiences broaden individuals’ momentary thought–action repertoires and, over time, build enduring personal resources. In the context of blended learning, diversified instructional activities—such as online self-directed exploration, multimedia resources, interactive forums, face-to-face discussions, and collaborative tasks—create repeated opportunities for students to experience positive emotions related to competence, autonomy, and social connection. These positive emotional experiences are not transient; rather, through sustained learning engagement, they gradually accumulate and transform into relatively stable psychological resources.

More concretely, the structural features of blended learning appear to map closely onto the core components of psychological capital. First, systematic and accessible online learning resources allow students to control learning pace and review content repeatedly, thereby strengthening mastery experiences and enhancing self-efficacy. Second, goal-oriented learning tasks and progressive assessments embedded in blended courses help students experience incremental success, which fosters hope by reinforcing both goal clarity and perceived pathways toward goal attainment. Third, timely feedback, iterative learning cycles, and opportunities to revise and improve performance cultivate resilience by normalizing challenge and recovery as integral parts of the learning process. Finally, the flexible integration of online and offline instruction helps students maintain a more optimistic outlook toward learning by reducing anxiety associated with rigid instructional structures and providing multiple avenues for success.

Importantly, the mediation analysis shows that learning engagement accounts for a substantial proportion of the effect of blended learning quality on psychological capital. This finding underscores that psychological capital is not enhanced simply by exposure to blended instructional environments, but rather through students’ active psychological involvement in learning. This insight aligns with prior research emphasizing engagement as a crucial psychological mechanism linking instructional design and learning outcomes, and highlights the importance of designing blended courses that genuinely stimulate students’ cognitive, emotional, and behavioral investment rather than merely incorporating technological elements.

### 5.2 Cultural contextualization: blended ideological and political courses within Chinese value systems

Beyond general learning mechanisms, the effects observed in this study must be understood within the specific cultural and educational context of China. Unlike generic blended courses, ideological and political courses are deeply embedded in value education and collective meaning construction. The present findings suggest that the psychological benefits of blended ideological and political courses are amplified by their alignment with Chinese cultural values emphasizing collectivism, social responsibility, and moral self-cultivation.

In traditional Chinese cultural philosophy, individual development is closely linked to collective well-being, as reflected in the ideal of progressing from “self-cultivation” to “family harmony,” “social governance,” and ultimately “world peace.” Blended ideological and political courses appear particularly well-suited to operationalize this value logic in contemporary educational settings. On the one hand, online instructional components often include narratives of national development, exemplary figures, and collective achievements, which can strengthen students’ sense of belonging and shared identity. On the other hand, offline classroom discussions and group-based learning activities encourage students to situate personal experiences and aspirations within broader social and national contexts.

This integration of individual learning and collective meaning may explain why learning engagement in ideological and political courses has a strong association with psychological capital. When students perceive their learning as contributing not only to personal success but also to social value, the psychological resources they develop—such as hope and optimism—are grounded in a more stable and transcendent meaning system. Prior studies have suggested that meaning-oriented educational experiences are particularly effective in fostering durable psychological resources (Fu, 2019; Fu, 2020; Luo and Xiao, 2020). The present study provides empirical support for this argument by demonstrating that blended ideological and political courses can simultaneously enhance engagement, psychological capital, and well-being.

Moreover, this culturally embedded mechanism distinguishes blended ideological and political education from many Western-oriented positive psychology interventions that focus primarily on individual achievement and personal happiness. In the Chinese context, psychological capital cultivated through value-oriented education may possess a stronger collective dimension, which could make it more resilient to external stressors and social uncertainty. This interpretation is consistent with findings by De Moortel et al. (2024), who emphasized the role of social context in shaping access to and returns from psychological capital.

### 5.3 Implications for cross-cultural positive psychology and educational practice in the digital era

The present findings offer important implications for cross-cultural dialogue in positive psychology and for understanding how formal curricula can function as scalable psychological interventions. Consistent with the foundational proposition of positive psychology that well-being can be cultivated through the systematic development of positive resources (Seligman, 2002; Csikszentmihalyi and Seligman, 2000; Gable and Haidt, 2005), this study confirms the central role of psychological capital in predicting students’ subjective well-being, and further demonstrates that psychological capital can be meaningfully shaped by structured educational experiences. In particular, the verified chain mediation pathway—blended learning quality → learning engagement → psychological capital → well-being—provides a process-based explanation for how pedagogical design and learning experience are translated into durable psychological resources and positive well-being outcomes.

From a theoretical standpoint, the most significant contribution of this study lies in the expansion and empirical validation of a localized positive psychology educational intervention model rooted in Chinese higher education. Although positive psychology originated in Western contexts, its constructs and interventions are increasingly recognized as culturally contingent and should be examined within specific sociocultural and institutional settings (Seligman and Csikszentmihalyi, 2014; Alex Linley et al., 2006; Seligman, 2019). By conceptualizing blended ideological and political courses as a systematic intervention context, this study connects “psychological capital” and “well-being”—two core constructs widely discussed in positive psychology—with the Chinese higher education mission of moral cultivation and value-oriented education. Importantly, the empirical model goes beyond the traditional research paradigm that treats the effectiveness of ideological and political courses primarily in terms of political identity or value internalization. Instead, it proposes and validates an integrated framework that links value guidance, technological empowerment, psychological development, and well-being enhancement, thereby offering a new lens through which the psychological functions of formal curricula can be understood.

These results also enrich cross-cultural positive psychology by highlighting the culturally embedded nature of well-being pathways. In many Western settings, psychological capital and happiness are often framed in relation to individual competence, personal success, and autonomous self-realization. In the Chinese context, however, students’ well-being tends to be grounded in the perceived unity between individual development and collective values, where meaning construction and value identity play a particularly salient role. The current findings suggest that blended ideological and political education may facilitate precisely such a meaning-oriented psychological process: students’ active engagement in value-infused learning experiences contributes to the accumulation of psychological capital, which then enhances subjective well-being. This culturally situated mechanism resonates with the argument that psychological resources are shaped by social structures and ideological contexts (Demerath et al., 2008) and that psychological capital may also reflect broader patterns of social inequality and contextual advantage (De Moortel et al., 2024). Therefore, the present study provides empirical material for cross-cultural dialogue by illustrating how positive psychological development can be promoted through a formal, value-oriented curriculum within a non-Western educational system.

Beyond theoretical significance, the study yields clear practical implications for educational reform and teaching design in the era of digitalization. Nevertheless, these practical implications should be interpreted with caution. Because the present study is based on cross-sectional survey data, the findings demonstrate perceived relationships among blended learning quality, engagement, psychological capital, and well-being rather than definitive causal effects of specific teaching reforms or policy interventions. Future research employing longitudinal designs, experimental interventions, or multi-wave data collection would provide stronger evidence for evaluating the long-term educational impact of blended ideological and political courses.

First, the findings provide empirical insights into how the digital transformation of ideological and political courses may contribute to students’ psychological development. The results indicate that improving the perceived quality of blended learning environments may foster learning engagement, which is associated with the development of psychological capital and well-being. However, such interpretations should be considered as potential implications rather than direct prescriptions for educational policy. Second, at the level of instructional practice, the results suggest that teachers may consider embedding psychologically supportive instructional strategies within blended course design. For example, teachers may cultivate self-efficacy by designing scaffolded online tasks that allow students to experience mastery, develop hope through challenging yet decomposable group projects, strengthen resilience through iterative feedback and revision opportunities, and enhance optimism by guiding students to adopt constructive attribution patterns in the face of setbacks (Lee Duckworth et al., 2005). Third, the measurement framework validated in this study may provide a useful diagnostic perspective for evaluating the psychological effectiveness of blended courses. At the same time, it is important to acknowledge that the current model was developed within the specific institutional and cultural context of ideological and political education in Chinese universities. Therefore, its applicability to other academic disciplines or educational systems should be examined cautiously. Future studies could test the model in different subject areas, cultural contexts, and educational systems to evaluate the broader generalizability of the proposed relationships.

Taken together, the present study suggests that positive psychology interventions need not be confined to individualized training programs or clinical contexts. Under appropriate instructional design, formal courses—especially those embedded in culturally meaningful value systems—can become an important institutional pathway for cultivating psychological capital and enhancing well-being in the digital age. Future research should continue to examine culturally differentiated mechanisms of psychological capital formation and well-being enhancement, and explore how variations in digital teaching design, course content, and student characteristics may shape the effectiveness of such curriculum-based psychological interventions.

## 6 Conclusion

This study verified the mechanism of the effect of blended ideological and political courses on college students’ psychological capital and happiness under the background of educational digitization through empirical analysis. Research has found that the quality of blended learning not only directly promotes students’ learning engagement, but also indirectly enhances their sense of well-being through the chain mediation of learning engagement and psychological capital. This conclusion reveals the complete pathway of “teaching experience psychological process happiness outcome,” providing a scientific basis for understanding the positive psychological benefits of ideological and political courses. On a theoretical level, this study constructed an integrated model that combines educational technology, positive psychology, and ideological and political education, expanding the localization application of positive psychology in educational contexts. At the practical level, the research results provide empirical support for optimizing the teaching design of blended ideological and political courses in universities, and suggest that educators should pay attention to stimulating students’ positive psychological resources through technological empowerment and teaching design. Future research can further explore the differentiated effects of different student groups, as well as the specific mechanisms of the interaction between course content and various dimensions of psychological capital.

Despite the theoretical and practical contributions of this study, several limitations should be acknowledged. First, this study employed a cross-sectional survey design based on students’ self-reported perceptions, which limits the ability to establish causal relationships among the variables. Although the structural model and mediation analysis provide evidence supporting the hypothesized relationships, the results should be interpreted as associations rather than definitive causal effects. Future research could adopt longitudinal or experimental designs to examine how changes in instructional practices influence students’ learning engagement, psychological capital, and well-being over time. Multi-wave data collection would also allow researchers to more rigorously test the temporal ordering of the sequential mediation model proposed in this study. Second, all variables in this study were measured using self-report questionnaires. Although statistical tests suggested that common-method bias was not a serious concern, the use of a single data source may still introduce potential measurement bias. Future studies could strengthen methodological rigor by incorporating multiple data sources, such as teacher evaluations, behavioral learning analytics, or observational measures of classroom engagement. Combining subjective and objective indicators would provide a more comprehensive understanding of the psychological processes involved in blended learning environments. Third, the study focused specifically on blended ideological and political courses within the Chinese higher education system. Such courses possess distinctive curricular goals related to value education, collective identity, and moral development, which may shape the way students interpret learning experiences and develop psychological resources. Therefore, the psychological mechanisms identified in this study may be partly influenced by the unique cultural and institutional context of ideological and political education. Future research could test the applicability of the proposed model in other academic disciplines—such as social sciences, engineering, or humanities—and across different educational systems to further explore the cultural and contextual boundaries of the findings.

## Data Availability

The original contributions presented in the study are included in the article/supplementary material, further inquiries can be directed to the corresponding author.
